# Propensity-adjusted 8-year outcomes following bioprosthetic aortic valve replacement: The influence of novel anticalcification technology

**DOI:** 10.1016/j.xjon.2025.101557

**Published:** 2025-12-15

**Authors:** Tsuyoshi Kaneko, Joseph E. Bavaria, Vinod H. Thourani, Douglas R. Johnston

**Affiliations:** a Division of Cardiothoracic Surgery, Washington University, St Louis, Mo; b Department of Cardiovascular Surgery, Jefferson Health, Philadelphia, Pa; c Department of Cardiovascular Surgery, Marcus Valve Center, Piedmont Heart Institute, Atlanta, Ga; d Division of Cardiac Surgery, Bluhm Cardiovascular Institute at Northwestern Medicine, Chicago, Ill

**Keywords:** aortic valve replacement, aortic valve disease, bioprosthetic valve, RESILIA tissue, long-term durability

## Abstract

**Objective:**

Aortic bioprostheses with RESILIA (Edwards Lifesciences) tissue technology have demonstrated robust outcomes through 7 years of follow-up. However, studies comparing RESILIA tissue valve outcomes to previous bioprosthetic valves are lacking. We sought to assess surgical aortic valve replacement outcomes in patients implanted with RESILIA tissue valves compared with non-RESILIA valves (PERIMOUNT Magna Ease with ThermaFix treatment; Edwards Lifesciences) at 8 years using data from 2 single-arm trials.

**Methods:**

The analysis consisted of 689 RESILIA patients from the Prospective, Non-Randomized, Multicenter Clinical Evaluation of Edwards Pericardial Bioprostheses With a New Tissue Treatment Platform (COMMENCE) Aortic trial and 258 non-RESILIA surgical aortic valve replacement patients from the Magna Ease postapproval study. To account for differences in baseline characteristics in these cohorts, stabilized inverse probability of treatment weighting with propensity score was used to analyze safety endpoints, including structural valve deterioration (SVD), reoperation due to SVD, all-cause reoperation, and all-cause mortality.

**Results:**

Mean age of the propensity score-adjusted cohort was 67 years, with a majority of male patients. After adjustment, all prespecified clinically relevant baseline variables were appropriately matched. At 8 years, the RESILIA cohort significantly outperformed non-RESILIA valves in freedom from reoperation (97.0% vs 90.5%; *P* = .0014), reoperation due to SVD (99.2% RESILIA vs 93.9% non-RESILIA; *P* = .0007), and SVD (99.3% vs 90.5%; *P* < .0001). Freedom from all-cause mortality was not statistically different between cohorts (83.3% RESILIA vs 81.3% non-RESILIA; *P* = .6332).

**Conclusions:**

In this propensity-adjusted patient population, surgical aortic valve replacement with this novel tissue treatment was associated with significantly lower rates of SVD, reoperation due to SVD, and all-cause reoperation at 8 years, with similar rates of all-cause mortality.

## Graphical Abstract

**Figure:**
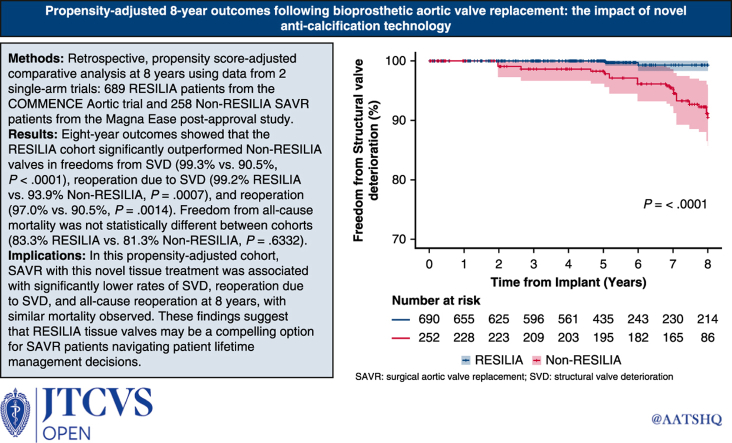

**Figure:**
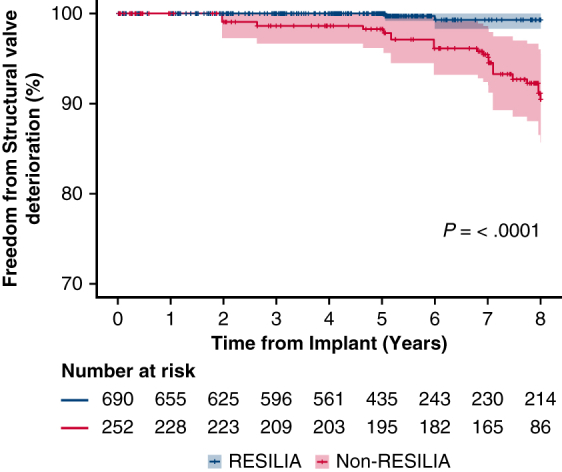
Freedom from SVD in RESILIA (Edwards Lifesciences) and non-RESILIA valves. 95% CI.

**Central Message**

At 8 years, RESILIA tissue valves (Edwards Lifesciences) significantly outperformed non-RESILIA valves in freedom from reoperation, reoperation due to SVD, and SVD.

**Perspective**

This study presents the longest follow-up and first long-term, propensity score-matched analysis comparing outcomes of RESILIA valves (Edwards Lifesciences) to a contemporary bioprosthesis. SAVR with the novel tissue was associated with less SVD, reoperation due to SVD, and all-cause reoperation at 8 years. Continued follow-up of RESILIA valves in the COMMENCE trial will further assess durability.

Aortic valve disease (AVD) is among the most treated valvular heart diseases in developed countries, and it is commonly associated with aging and subsequent degenerative-calcific processes leading to stenosis.^1^^,^^2^ Surgical aortic valve replacement (SAVR) is an established treatment for AVD requiring intervention. The choice of bioprosthetic versus mechanical valves is balanced by the risk of structural valve deterioration (SVD) in bioprosthetic valves, as opposed to lifelong anticoagulation therapy in mechanical valves.^3^^,^^4^ The 2020 American College of Cardiology/American Heart Association guidelines recommend mechanical valves for patients younger than age 50 years, whereas bioprosthetic valves have a Class II recommendation in patients aged 65 years and older.^3^ However, with patient preference being the Class I recommendation, including individualized discussion of risks, the majority of patients choose bioprosthetic valves to avoid anticoagulation and accept the risk of SVD.^3^ A recent Society of Thoracic Surgeons database analysis verified this by showing that bioprosthetic valve use in patients undergoing SAVR between ages 40 and 75 years increased to 90.2% from 2008 to 2019, whereas mechanical valve utilization decreased to 9.8%.^5^ Hence, there is a critical need for more advanced bioprosthetic valve technology and more long-term clinical investigation to address these tradeoffs.

The novel RESILIA bovine pericardial tissue technology (Edwards Lifesciences) was designed with a proprietary stable-capping process to mitigate free-aldehyde binding to calcium, potentially improving valve durability.^6^ In addition, glycerolization of this tissue displaces water, thereby minimizing exposure to glutaraldehyde, allowing for dry storage of the valve. RESILIA tissue valves performed significantly better than traditional tissue valves in animal studies and have shown promising results with clinically stable hemodynamics and 99.3% freedom from SVD reported through 7 years of follow-up.^7^, ^8^, ^9^, ^10^

SVD in previous generations of bioprosthetic valves is rare before 5 years postimplant.^11^ SVD has been observed in the 5- to 7-year time frame, with durability declining more rapidly after that period.^12^ Therefore, the objective of this study was to perform a propensity score-matched analysis comparing longest-available outcomes of SAVR with RESILIA tissue valves versus a widely used previous generation bioprosthesis, the Magna Ease valve with ThermaFix tissue treatment (Edwards Lifesciences).^13^

## Methods

### Study Design

Two prospective, single-arm studies of patients undergoing SAVR served as the basis for this nonrandomized ad hoc comparative analysis (Table 1). In brief, patient-level data from both trials were utilized with propensity score adjustment applied to account for differences in baseline characteristics between the cohorts. Valves from the 2 trials were identical in tissue annulus diameter (19, 21, 23, 25, 27, and 29 mm) and all other structural characteristics, except for tissue treatment.

**Table 1:** Study design for valve cohorts (RESILIA [Edwards Lifesciences] vs non-RESILIA valve recipients) in the Prospective, Non-Randomized, Multicenter Clinical Evaluation of Edwards Pericardial Bioprostheses With a New Tissue Treatment Platform (COMMENCE) Aortic study and Magna Ease Aortic Valve study

Trial characteristic	RESILIA tissue valve: COMMENCE-Aortic study	Non-RESILIA tissue valve: Magna Ease Aortic Valve study
ClinicalTrials.gov registration	NCT01757665	NCT01171625
FDA status	Investigational device exemption trial	Postapproval study
Design	Prospective, single arm	Prospective, single arm
Intervention	SAVR with bovine pericardial bioprosthesis with RESILIA tissue treatment, on PERIMOUNT∗ platform	SAVR with Magna Ease bovine pericardial bioprosthesis with ThermaFix treatment, on PERIMOUNT platform
Locations	Multicenter (United States, Europe)	Multicenter (United States, Canada, Europe)
No. of patients	689	258
Relevant publications	Puskas and colleagues,^14^ 2017 Bavaria and colleagues,^15^ 2023 Beaver and colleagues,^7^ 2024	Tsui and colleagues,^13^ 2022
Safety event definitions	Akins and colleagues,^16^ 2008 Clinical events committee adjudicated	Akins and colleagues^16^ 2008 Clinical events committee adjudicated
Efficacy end point definitions	NYHA functional class Valve hemodynamic performance, with echocardiograms assessed by independent core lab	NYHA functional class Valve hemodynamic performance, with echocardiograms assessed by study sites only

*FDA*, Food and Drug Administration; *SAVR*, surgical aortic valve replacement; *NYHA*, New York Heart Association.

∗ Edwards Lifesciences.

### Patient Eligibility

Inclusion criteria for the 2 cohorts were similar to each other and have been reported previously.^13^^,^^14^ In general, adult patients undergoing SAVR with or without concomitant procedures (but excluding patients requiring replacement/repair of other heart valves) were eligible for participation in each of the studies. All study participants provided informed written consent for the publication of their study data. Institutional review board approvals for both trials can be found in Appendix E1.

### Outcomes

Safety endpoints included SVD, reoperation due to SVD, reoperation for any cause, mortality, valve-related mortality, stroke, major bleeding, nonstructural valve dysfunction, endocarditis, and thromboembolism. Safety events for both studies were defined by Akins criteria,^16^ adjudicated by their respective clinical events committees and assessed over the full 8-year follow-up period. In brief, SVD was defined as dysfunction or deterioration involving the operated valve (exclusive of infection or thrombosis), as determined by reoperation, autopsy, or clinical investigation. Nonstructural valve dysfunction was defined as any abnormality not intrinsic to the valve itself that results in stenosis or regurgitation of the operated valve or in hemolysis. Valve hemodynamic outcomes were assessed by echocardiography core laboratory in reconsented patients from the RESILIA cohort,^7^ whereas site-reported echocardiography results were recorded for the non-RESILIA cohort, precluding direct comparison of hemodynamic results. Mean gradient and effective orifice area (EOA) within each cohort were summarized as the mean ± SD at each available time of assessment.

### Statistical Analysis

Stabilized inverse probability of treatment weighting with propensity score was used rather than a 1:1 matched analysis to minimize bias while retaining all patients from the original study populations. The propensity score was calculated based on the following prespecified clinically meaningful baseline variables: age, sex, New York Heart Association (NYHA) class, body mass index, transient ischemic attack/cerebrovascular accident, coronary artery disease, renal failure/insufficiency, diabetes, prior pacemaker implant, coronary artery bypass graft procedure, history of myocardial infraction, chronic obstructive pulmonary disease, moderate/severe mitral regurgitation, and prior aortic valve intervention.^11^ The probability of a patient being assigned to the relevant treatment arm given the observed baseline characteristics was then estimated using logistic regression.^17^ The inverse probability of treatment weighting for each patient was calculated as the inverse of the probability of that patient receiving the observed treatment, as estimated by propensity score. To address the potential issue of extreme weights that may distort estimates of treatment effects, the inverse probability of treatment weighting was stabilized for all adjusted analyses. The weighted Kaplan-Meier time-to-event analysis and log-rank test were used to compare safety events between treatment groups.

To assess balance between the 2 cohorts with respect to baseline variables, the absolute standardized mean difference was computed. In alignment with prior literature, an absolute standardized mean difference <0.25 was utilized to indicate no statistically significant difference between cohorts.^18^^,^^19^ Summary statistics for both cohorts are presented as percentages for categorical variables and as means ± SD for continuous variables. All analyses were performed in SAS version 9.4 (SAS Institute Inc).

## Results

The study population consisted of 947 patients who underwent SAVR: 689 with novel RESILIA tissue valves and 258 using an earlier-generation bovine pericardial tissue valve on the same platform (non-RESILIA; PERIMOUNT Magna Ease with ThermaFix treatment). Baseline characteristics for patients in these trials have been previously published^7^^,^^13^ and are shown in Table E1. In brief, RESILIA patients had a mean age of 66.9 ± 11.6 years, whereas non-RESILIA patients were slightly older, with a mean age of 68.5 ± 8.8 years. Before propensity score adjustment, more patients in the RESILIA cohort had renal failure/insufficiency (8.6% vs 2.7% for non-RESILIA) and coronary artery disease (55.9% vs 41.1% for non-RESILIA). Patients in both trials were predominantly men (71.8% RESILIA, 64.7% non-RESILIA) and in NYHA class I or II (73.7% RESILIA, 67.5% non-RESILIA). Valve size distributions were similar between the 2 studies (*P* = .384). Further, 22.2% and 20.2% of RESILIA and non-RESILIA participants, respectively, were implanted with either 19- or 21-mm valves.

The 2 cohorts were well balanced for patient characteristics after propensity score adjustment using prespecified criteria (Table 2). The mean age of the propensity score-adjusted cohort was 67 years. The majority of patients were men and 28% had heart failure (NYHA class III-IV). More than half of patients in both cohorts had isolated SAVR, with the remaining patients split between SAVR plus coronary artery bypass graft and SAVR plus other procedures.

**Table 2:** Characteristics of patients receiving RESILIA valves (Edwards Lifesciences) or non-RESILIA valves after propensity score adjustment

Patient characteristic	RESILIA tissue valves (n = 689)	Non-RESILIA tissue valves (n = 258)	Absolute standardized mean difference∗
Age (y)	67.4 ± 11.4	67.5 ± 9.1	0.0047
Female (%)	30.2	33.3	0.0660
BMI	29.7 ± 5.7	29.8 ± 6.0	0.0167
Comorbidities (%)			
Heart failure†	27.7	28.9	0.0282
Diabetes mellitus	26.6	25.4	0.0275
Renal failure/insufficiency	6.9	4.0	0.1277
Chronic obstructive pulmonary disease	13.3	13.8	0.0147
Coronary artery disease	51.8	51.1	0.0151
Prior stroke/TIA	7.6	5.8	0.0737
Prior myocardial infarction	7.0	5.7	0.0551
Moderate/severe mitral valve regurgitation	2.7	2.5	0.0084
Prior procedures (%)			
Prior aortic valve intervention	3.3	2.4	0.0518
Prior CABG	2.8	1.3	0.1115
Prior pacemaker	2.4	2.7	0.0195

Values are presented as mean ± SD unless otherwise noted. *BMI*, Body mass index; *TIA*, transient ischemic attack; *CABG*, coronary artery bypass graft.

∗ Propensity score-adjusted cohorts were considered sufficiently similar for a given characteristic if absolute standardized mean difference <0.25.^18^^,^^19^

† New York Heart Association functional class III or IV.

**Figure 1:**
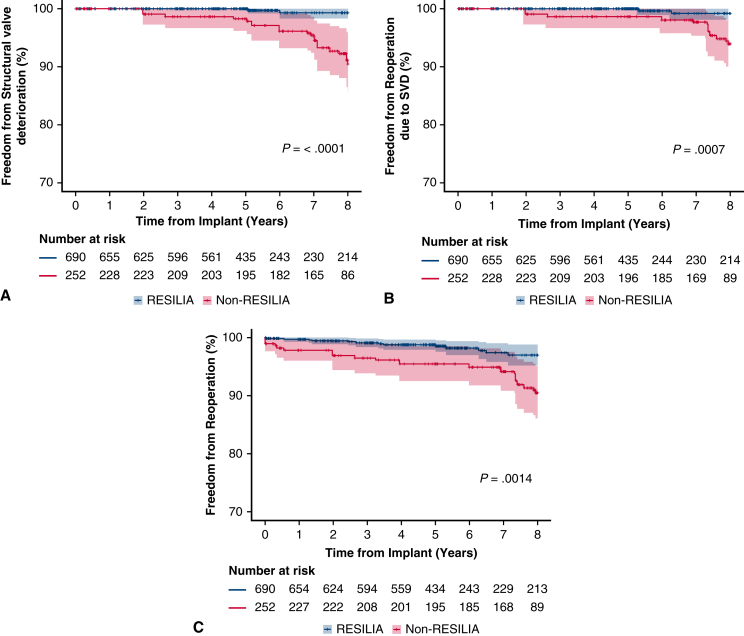
Freedom from structural valve deterioration (SVD) (A), reoperation due to SVD (B), and reoperation for any cause (C). 95% CI.

Through 8 years, 99.3% of patients in the RESILIA cohort were free from SVD compared with 90.5% in the non-RESILIA cohort, which was statistically significant in the propensity score-adjusted analysis (Figure 1, *A*) (*P* < .0001). Similarly, freedom from reoperation due to SVD (99.2% vs 93.9%; *P* = .0007) (Figure 1, *B*) and all-cause reoperation (97.0% vs 90.5; *P* = .0014) (Figure 1, *C*) favored the RESILIA cohort at 8 years.

**Figure 2:**
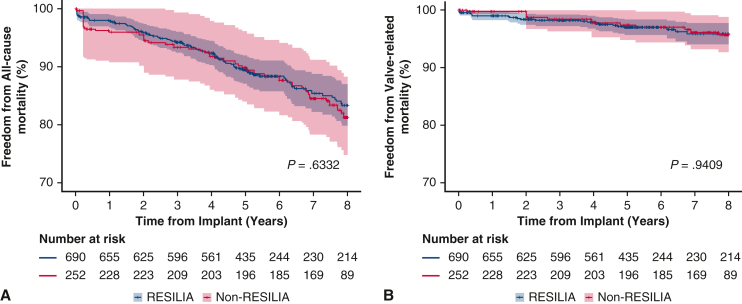
Freedom from all-cause mortality (A) and valve-related mortality (B). 95% CI.

Two SVDs occurred in the RESILIA cohort, whereas 16 were observed in the non-RESILIA cohort. Of the reoperations due to SVD in the RESILIA cohort, both were related to stenosis and occurred at 5.3 and 6.3 years, respectively. Of the 16 SVDs in the non-RESILIA cohort, 12 were secondary to stenosis, whereas the remaining causes included 1 subject with mixed disease and 3 with regurgitation. Ten reoperations due to SVD were observed in the non-RESILIA cohort with events occurring between 2.0 and 7.9 years. Five of the remaining SVDs in the non-RESILIA cohort did not undergo reintervention, and 1 patient died (Table 3).

**Table 3:** Cause and timing of structural valve deterioration (SVD)

Cohort	SVD type	Mode	Outcome	Time to reoperation (y)	Time to SVD (y)
RESILIA∗	Calcification	Stenosis	ViV	5.3	5.1
RESILIA	Restricted leaflet motion of uncertain etiology	Stenosis	Explant	6.3	6.0
Non-RESILIA	Calcification	Stenosis	Explant	2.0	2.0
Non-RESILIA	Stenosis	Stenosis	Explant	2.6	2.6
Non-RESILIA	Calcification	Stenosis	ViV	6.0	6.0
Non-RESILIA	Mild leaflet restriction	Regurgitation	ViV	6.9	6.9
Non-RESILIA	Stenosis	Stenosis	ViV	7.3	6.8
Non-RESILIA	Calcification	Stenosis	ViV	7.3	7.0
Non-RESILIA	Stenosis	Stenosis	Explant	7.4	7.0
Non-RESILIA	Severe AI/regurgitation	Regurgitation	ViV	7.6	7.5
Non-RESILIA	Stenosis	Stenosis	ViV	7.9	7.0
Non-RESILIA	Stenosis and insufficiency	Mixed	ViV	7.9	7.7
Non-RESILIA	Stenosis	Stenosis	ViV	–	5.0
Non-RESILIA	Stenosis	Stenosis	Not reintervened	–	6.0
Non-RESILIA	Stenosis: mild	Stenosis	Not reintervened	–	8.0
Non-RESILIA	Stenosis	Stenosis	Not reintervened	–	8.0
Non-RESILIA	Regurgitation	Regurgitation	Not reintervened	–	5.2
Non-RESILIA	Stenosis	Stenosis	CHF/death	–	4.6

*ViV*, Transcatheter valve-in-valve replacement; *AI*, aortic insufficiency; *CHF*, congestive heart failure.

∗ Edwards Lifesciences.

Female sex and valve size are also important factors to consider in evaluating valve durability. Of the 2 SVDs in the RESILIA cohort, both occurred in female participants implanted with 21-mm and 25-mm trial valves, as previously reported.^7^ Of the 16 SVDs in the non-RESILIA cohort, 7 were in women; of these, 1 was implanted with a 19-mm valve, 2 with 21-mm valves, and 4 with 23-mm valves. Among women who experienced SVD, similar proportions had 19- or 21-mm valves across cohorts: 50% in the RESILIA group and 43% in the non-RESLIA group.

Overall survival at 8 years after SAVR was not statistically different between the 2 valve cohorts (83.3% vs 81.3%; *P* = .6332) (Figure 2, *A*). Freedom from valve-related mortality was more than 95% in both cohorts with no significant difference observed (*P* = .9409) (Figure 2, *B*). Additional safety events following SAVR were assessed in the propensity score-adjusted cohorts and are summarized in Table 4. No significant differences were observed between cohorts for stroke, endocarditis, or thromboembolism. Compared with the previous generation valve, the RESILIA cohort had significantly lower risk of major bleeding (freedom from major bleeding: 90.4% vs 85.3%; *P* = .0177) and greater freedom from nonstructural valve dysfunction (99.1% vs 97.9%; *P* = .0296).

**Table 4:** Safety events throughout the follow-up period for propensity score-adjusted cohorts of patients receiving a RESILIA tissue valve (Edwards Lifesciences) or a non-RESILIA tissue valve

Event	RESILIA tissue valve (n = 689)	Non-RESILIA tissue valve (n = 258)	Log-rank *P* value between cohorts
All-cause mortality	83.3	81.3	.6332
Valve-related mortality	95.9	95.7	.9409
Stroke	92.8	93.2	.9020
Thromboembolism	88.5	85.5	.2823
Major bleeding	90.4	85.3	**.0177**
Endocarditis	97.4	97.0	.6906
SVD	99.3	90.5	**<.0001**
Nonstructural valve dysfunction	99.1	97.9	**.0296**
Reoperation due to SVD	99.2	93.9	**.0007**
Reoperation	97.0	90.5	**.0014**

Values are presented as (%). *SVD*, Structural valve deterioration. *P*-values below .05 (bolded) are considered statistically significant.

**Figure 3:**
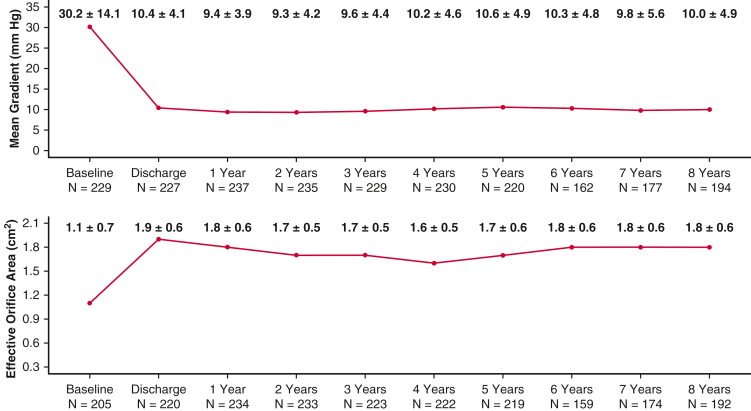
Hemodynamic parameters of patients in the RESILIA valve (Edwards Lifesciences) cohort: Echocardiograph-derived mean gradients (mm Hg) and effective orifice area (cm^2^).

Clinically stable mean gradient and EOA were observed through 8 years with the RESILIA tissue valve (Figure 3), consistent with the present findings of favorable data on SVD. Direct comparison of hemodynamics data was not possible due to a lack of core lab-assessed data in the non-RESILIA cohort. Site-reported data in this cohort also indicated broadly stable hemodynamics and are presented in Table E2.

## Discussion

This study represents the first long-term, propensity-score matched analysis with the longest available clinical follow-up directly comparing the novel tissue technology (RESILIA) to PERIMOUNT Magna Ease with ThermaFix treatment, and it provides several key findings. First, RESILIA valves demonstrated improved durability compared with the previous tissue valve, with lower risk of SVD and reoperation at 8 years. Second, freedom from mortality was similar between the 2 groups. Lastly, the echocardiographic data showed clinically stable mean gradient and EOA. The observed stable hemodynamics and very low incidence of clinically adjudicated SVD support the potential for increased valve longevity with RESILIA tissue.

Careful consideration to patient lifetime management in AVD has become more critical with expanding treatment options, new indications, and the possibility of future valve interventions, such as transcatheter valve-in-valve replacement.^20^ Potentially delaying the need for reintervention due to SVD would be a significant advance for bioprosthetic valves, which may influence how valves are selected, particularly in younger patients. Durability benefits became evident in the 7- to 8-year timeframe in our study, highlighting the importance of clinical characterization of bioprosthetic valves through extended follow-up.

The findings of the present study are consistent with the excellent outcomes from recent subanalyses of RESILIA tissue outcomes in patients with aortic regurgitation and bicuspid aortic valve.^15^^,^^21^^,^^22^ Prior studies have performed comparisons to RESILIA tissue with consistent outcomes to those of the present study reported, albeit at earlier follow-up durations. Bartus and colleagues^23^ compared SVD-related hemodynamic valve deterioration in RESILIA versus non-RESILIA tissue valves through 5 years of follow-up and found reduced deterioration in RESILIA tissue valves relative to a cohort of predominantly Carpentier-Edwards PERIMOUNT valves (1.0 vs 4.8% in propensity-matched cohorts). Other comparative studies have been limited to early-term follow-up (1-3 years) and have not demonstrated a significant difference in durability outcomes.^24^^,^^25^ Taken together, these studies add to the growing body of evidence supporting use of this novel tissue in SAVR.

Strengths of the current analysis include the longest follow-up to date of the RESILIA novel tissue valve in comparison to a widely used previous generation valve. The RESILIA group consisted of patients implanted with a Magna Ease valve supplemented with RESILIA, whereas the non-RESILIA group consisted of Magna Ease valves with ThermaFix treatment. As a result, this study is the first true clinical investigation of RESILIA tissue treatment relative to contemporary ThermaFix tissue treatment, the results of which speak to the potential of improved valve longevity and reduced reintervention with RESILIA tissue valves. Propensity score adjustment was used to ensure that the 2 cohorts were comparable at baseline with a prespecified list of covariates utilized to minimize bias. In addition, the inclusion of 2 prospective clinical trials led to better-quality data and more clinical detail than in most retrospective clinical studies.

Although this study presents important findings, this analysis does have limitations to be considered. This study compares outcomes from 2 distinct clinical trials. Though broadly similar in design and patient eligibility, these trials were not designed to be directly compared, which may result in potential biases and confounding factors such as use of different clinical events committees. Differences in patient characteristics may persist despite the propensity score adjustment; in particular, the variables used for matching did not include etiology, valve size, or concomitant procedures (although the trials both excluded replacement or repair on other valves). Further, enrollment for the Magna Ease postapproval study initiated in 2007, whereas the Prospective, Non-Randomized, Multicenter Clinical Evaluation of Edwards Pericardial Bioprostheses With a New Tissue Treatment Platform (COMMENCE) trial began in 2012. At the time of trial initiation in consultation with the Food and Drug Administration, the Akins definition was selected to evaluate SVD because it was considered a standard method by the clinical community.^16^ However, this definition may underestimate SVD compared with more recent definitions such as Valve Academic Research Consortium criteria that account for hemodynamic compromise over time.^26^ A key finding of the study was the clinically stable hemodynamics observed in the RESILIA cohort. Given that the non-RESILIA cohort did not have core lab-adjudicated echocardiographic data, this limited the ability to perform either a direct comparison of hemodynamic outcomes or a subanalysis of SVD per Valve Academic Research Consortium criteria. The primary endpoints of this study were safety outcomes that addressed the key question of bioprosthetic valve durability. Although this is now the longest follow-up for RESILIA tissue valves, additional follow-up beyond 8 years is warranted and will be addressed as COMMENCE trial patients complete 10 years of follow-up.

## Conclusions

In this propensity score-adjusted patient population, SAVR with RESILIA tissue technology was associated with both clinically stable hemodynamics and significantly lower rates of SVD, reoperation due to SVD, and reoperation at 8 years compared with a widely used bioprosthetic valve. These data suggest that RESILIA tissue valves may be a compelling option for patients undergoing SAVR as they navigate challenging lifetime management considerations that aim to maximize life expectancy while balancing valve durability. Continued follow-up of RESILIA valves through 10 years in the COMMENCE trial will be influential to reaffirm long-term durability of these valves.

## Conflict of Interest Statement

Dr Kaneko is an advisor for Edwards Lifesciences and Abbott Laboratories and a consultant for Medtronic, Johnson & Johnson, Anteris, and 4C Medical. Dr Bavaria is a consultant for Edwards Lifesciences, Abbott, Medtronic, Terumo Aortic, and Artivion. Dr Thourani does consulting or research for Abbott Vascular, Artivion, Atricure, Boston Scientific, Croivalve, Edwards Lifesciences, Highlife, Innovalve, Jenavalve, Medtronic, and Trisol. Dr Johnston is an advisor for Abbott Labs, Edwards Lifesciences, Terumo Cardiovascular, Livanova Inc, and HD Medical.

The *Journal* policy requires editors and reviewers to disclose conflicts of interest and to decline handling or reviewing manuscripts for which they may have a conflict of interest. The editors and reviewers of this article have no conflicts of interest.

## Figures and Tables

**Table E1:** Characteristics of patients receiving RESILIA valves (Edwards Lifesciences) or non-RESILIA valves before propensity score adjustment

	RESILIA tissue valves (n = 689)	Non-RESILIA tissue valves (n = 258)	Absolute standardized mean difference∗
Age (y)	66.9 ± 11.6	68.5 ± 8.8	0.1549
Female (%)	28.2	35.3	0.1533
BMI	30.0 ± 5.7	28.9 ± 5.6	0.1863
Comorbidities (%)			
Heart failure†	26.3	32.5	0.1379
Diabetes mellitus	28.4	22.1	0.1466
Renal failure/insufficiency	8.6	2.7	0.2557
Chronic obstructive pulmonary disease	14.2	11.2	0.0896
Coronary artery disease	55.9	41.1	0.2993
Prior stroke/TIA	8.6	5.8	0.1066
Prior myocardial infarction	7.8	5.0	0.1142
Moderate/severe mitral valve regurgitation	2.9	1.9	0.0628
Prior procedures (%)			
Prior aortic valve intervention	4.2	0.8	0.2216
Prior CABG	3.6	0.8	0.1954
Prior pacemaker	3.2	0.4	0.2128

Values are presented as mean ± SD unless otherwise noted. *BMI*, Body mass index; *TIA*, transient ischemic attack; *CABG*, coronary artery bypass graft.

∗ Propensity score-adjusted cohorts were considered sufficiently similar for a given characteristic if absolute standardized mean difference <0.25.^18^^,^^19^

† Defined as New York Heart Association functional class III or IV.

**Table E2:** Hemodynamic parameters of patients in the cohort who did not receive a RESILIA valve (Edwards Lifesciences) (site-reported data, not propensity score-adjusted)

Time of assessment	Mean systolic gradient, (mm Hg)	Effective orifice area (cm^2^)
Baseline	42.5 (44.4)	0.9 (0.27)
Discharge	13.8 (5.56)	1.8 (0.55)
3-6 mo	12.3 (4.99)	1.7 (0.43)
1 y	12.2 (4.97)	1.7 (0.41)
2 y	13.3 (5.50)	1.6 (0.44)
3 y	13.6 (5.37)	1.6 (0.34)
4 y	13.1 (5.38)	1.6 (0.46)
5 y	14.0 (5.73)	1.6 (0.39)
6 y	13.7 (6.88)	1.6 (0.44)
8 y	14.1 (7.90)	1.6 (0.74)

Values are presented as n (%).

**Appendix E1:** Magna Ease postapproval study and Prospective, Non-Randomized, Multicenter Clinical Evaluation of Edwards Pericardial Bioprostheses With a New Tissue Treatment Platform (COMMENCE)-Aortic study institutional review board/ethics committee (IRB/EC) approvals.

Site	Site name Address	Principal investigator	IRB/EC name Address	IRB/EC approval date	Project ID approval reference
006	Medizinische Hochschule Carl-Neuberg-Straße 1, Hannover D-30625 Germany	Axel Haverich	Medizinische Hochschule Hannover Ethics Committee Carl-Neuberg-Straße 1, Hannover, 30,625 Germany	20-Aug-08	#5083
069	Papworth Hospital (NHS Foundation Trust) Papworth Everard, Cambridge CB23 3RE UK	Dr Steven Tsui	NHS National Research Ethics Service Victoria House - Capital Park Fulbourn - Cambridge CB21 5XB, UK	30-May-08	#08/H0304/34
096	University Hospital Puerta de Hierro San Martin de Porres 4, Madrid, CP-28035 Spain	Dr Santiago Serrano- Fiz	Comite Etico de Investigacion Clinica Arez VI 28,222 Madrid, Spain	26-Feb-08 (conditional approval) 25-Mar-08 (full approval)	#892
355	German Heart Center Lazarettstraße 36, München, 80,636 Germany	Prof Dr Rüdiger Lange	Technische Universität München Fakultät für Medizin Ethikkommission Ismaninger Str 22, 81,675 München, Germany	04-Dec-07	#1913/07
376	Universitäsklinik für Chirurgie, Anichstrasse 34 A-6020 Innsbruck, Austria	Dr med Orest Chevtchik	EC of the Medical University Innsbruck Geschäflsste Innrain 43/1, Stock A-6020, Innsbruck, Austria	29-Jan-08	#EK 1 25.09.07
467	Blackpool Victoria Hospital Lancashire Cardiac Centre Whinney Heys Rd, Blackpool FY3 8NP UK	Dr Joseph Zacharias	NHS Foundation Trust Research and EC Shinney Heys Rd, Blackpool Lancashire, FY3 8NR UK	23-Jul-08	#08/H0304/34
012	University of British Columbia, St Paul's Hospital 1081 Burrard St, Vancouver, BC V6Z 1Y6 Canada	James Abel, MD	UBC-PHC Research Ethics Board 11th Floor Hornby Site, SPH - c/o 1081 Burrard St, Vancouver, BC V6Z 1Y6 Canada	24-Jun-11	#H11-00994
214	Sunnybrook Health Science Center 2075 Bayview Ave, Toronto, ON M4N 3M5 Canada	Gideon Cohen, MD	Sunnybrook Health Sciences Center Research Ethics Board Sunnybrook Campus 2075 Bayview Ave, Room C819, Toronto, ON M4N 3M5 Canada	21-Feb-08	#382-2007
088	Providence Heart & Vascular Institute 9205 SW Barnes Rd, Portland, OR 97225	Julia Swanson, MD	Providence Heath & Services IRB 5251 NE Glisan, 3rd Floor, Portland, OR 97213	28-Jul-10	#10-063B
488	Mercy General Hospital 3941 J St, Sacramento, CA 95819	Allen Morris, MD	Mercy General Hospital IRB/Dignity Health IRB 3400 Data Dr, Rancho Cordova, CA 95670	17-Nov-11	#033588
549	Morristown Memorial Hospital 100 Madison Ave, Morristown, NJ 09762	John Brown, MD	Atlantic Health IRB PO Box 1905, Morristown, NJ, 07,962-1905	07-Jan-10	#R09-12-004 (455,058)
551	Cooper University Hospital 3 Cooper Plaza, Camden, NJ 08103	Michael Rosenbloom, MD	The Cooper Health System IRB 3 Cooper Plaza, Suite 504, Camden, NJ 08103	05-Feb-10	#09-190EX
575	St Marks Hospital 1200 E 3900 S, Salt Lake City, UT 84124	Gilbert R Schorlemmer, MD	Mountain Star St Mark's Hospital IRB 1200 E 3900 S, Salt Lake City, UT 84124	21-Jan-10	STMH#0221
576	University Medical Center 1501 N Campbell Ave, Tucson, AZ 85724	Michael Moulton, MD	Western IRB 1019 39th Ave, SE, Suite 120, Puyallup, WA 98374-2115	19-Apr-11	#20110574

COMMENCE IRB approvals					
083	AdventHealth Orlando 601 E Rollins St, MB #99 Orlando, FL 32803	Kevin Accola, MD	AdventHealth IRB Orlando 901 N Lake Destiny Rd, Suite 400, Maitland, FL 32751	24-Jan-14	Reference #: 0530, Project #: 507,204-4
169	Mount Sinai Hospital Icahn School of Medicine 1190 Fifth Ave, 2nd Floor, New York, NY 10029	David Adams, MD	Icahn School of Medicine at Mount Sinai One Gustave L. Levy Place, Box 1081, New York, NY 10029	27-Aug-13	Project #: HS #: 13-00479 GCO #: 13-1233(0001) (01)
254	Hospital of the University of Pennsylvania 6 Silverstein Pavilion, 3400 Spruce St, Philadelphia, PA 19104 Penn Presbyterian Med Ctr, 38th and Powelton Ave, Philadelphia, PA 19104 Research office location: University of Pennsylvania Office of Research Services P 221 Franklin Building, 3451 Walnut St, Philadelphia, PA 19104-6205	Joseph Bavaria, MD	Western IRB 1019 39th Ave, SE Suite 120, Puyallup, WA 98374-2115	24-Mar-14	Protocol #: 819,477
347	University of Florida Shands Healthcare 1600 SW Archer Rd, Room NG-33, Gainesville, FL 32610	Thomas Beaver, MD Previous PI: Charles Klodell, MD	Western IRB 1019 39th Ave, SE, Suite 120, Puyallup, WA 98374-2115	14-Feb-13	Western IRB Project #: 20,130,138; Work Order #: 1-762447-1
217	University of Utah Hosp Health Sciences Center 30 N 1900, SOM 3C-127 Salt Lake City, UT 84132 Research office location: 50 N Medical Dr, Salt Lake City, UT 84132	Craig H. Selzman, MD Previous PI: David Bull, MD	IRB University of Utah 75 S 2000 E RAB, Room 211, Salt Lake City, UT 84112	17-Sep-14	IRB_00072440
319	Ohio State University 1960 Kenny Rd Columbus, OH 43210 Research office location: David Heart and Lung Research Institute, 473 W 12th Ave, Suite 105, Columbus, OH 43210	Jovan Bozinovski, MD Previous PIs Jefferson Lyons, MD, Juan Crestanello, MD, and Robert Higgins, MD	Western IRB 1019 39th Ave, SE, Suite 120, Puyallup, WA 98374-2115	09-Apr-14	Western IRB Project #: 20,130,138, Work Order #: 1-831664-1
180	University of Maryland School of Medicine 110 S Paca St, 7th Floor, Baltimore, MD 21201	Bartley Griffith, MD Previous PI: James S. Gammie, MD	University of Maryland, Baltimore IRB 620 W Lexington St, 2nd Floor, Baltimore, MD 21201	14-Mar-13	Project #: HP-00054452
479	New York Presbyterian Weill Cornell Medical College 525 E 68th St, New York, NY 10065	Leonard Girardi, MD	Weill Cornell Medical College Division of Research Integrity Research & Sponsored Programs 407 E 61st St, First Floor, 1300 York Ave, Box 89, New York, NY 10065	21-May-13	Protocol #: HP-00054452
656	Emory University Hospital 550 Peachtree St, NE, MOT, 6th Floor-CRU, Atlanta, GA 30308 St Joseph's Hospital 5665 Peachtree Dunwoody Rd, Atlanta, GA 30342	Michael Halkos, MD Previous PI: John Puskas, MD	Emory University IRB 1599 Clifton Rd, 5th Floor, Atlanta, GA 30322	18-Feb-13	IRB00062456
085	St Vincent's Hospital 10,590 N Meridian St, Suite 105 Indianapolis, IN 46290	David Heimansohn, MD	St Vincent Hospital IRB Research & Regulatory Affairs 8402 Harcourt Rd Suite 120, Indianapolis, IN 46260	14-Nov-12	Protocol #: R2012-1461
001	Cleveland Clinic 9500 Euclid Ave, JJ5, Cleveland, OH 44195	Douglas Johnston, MD Previous PI: Lars Svensson, MD, PhD	Cleveland Clinic IRB 9500 Euclid Ave, Cleveland, OH 44195	12-Apr-13	IRB #:13-324
529	Missouri Baptist Medical Center 3009 N Ballas Rd, Suite 360C St Louis, MO 63131 Research office location: 3015 N Ballas Rd, St Louis, MO 63131	Nicholas Kouchoukos, MD	Missouri Baptist Medical Center IRB 3015 N Ballas Rd, St Louis, MO 63131	21-Jan-14	MBMC study #: 1007
682	Sutter Institute for Medical Research Sacramento Cardiovascular Surgeons Buhler Specialty Pavilion, 2800 L St, Suite 260, Sacramento, CA 95816 Research office location: Sutter Medical Center, 2825 Capitol Ave, Sacramento, CA 95816	Michael Ingram, MD Previous PI: James Longoria, MD	Western IRB 1019 39th Ave, SE, Suite 120, Puyallup, WA 98374-2115	27-Jun-14	Western IRB Protocol #: 20,130,138, Work Order #: 1-841810-1
064	Stanford University Research Management Group 3172 Porter Dr, Palo Alto, CA 94304 Research office location: CVRB, 2nd Floor, 300 Pasteur Dr, Stanford, CA 94305	Craig Miller, MD	Research Compliance Office, Stanford University 3000 El Camino Real, Five Palo Alto Square, 4th Floor, Palo Alto, CA 94306	28-Jan-14	Protocol ID: 29,276, IRB #: 351
407	Pinnacle Health at Harrisburg Hospital 111 S Front St Harrisburg, PA 17101 Research office location: Pinnacle Health Cardiovascular Institute 1000 N Front St, Wormleysburg, PA 17043	Mubashir Mumtaz, MD	UPMC Pinnacle IRB 307 S Front St, Press Hall, First Floor, Harrisburg, PA 17104	11-Feb-14	PHH #: 13-065
689	Michael E. DeBakey VA Medical Center and Houston VA Research & Education Foundation Inc 2002 Holcombe Blvd, Building 100, Room 5B-170C Houston, TX 77030	Lorraine Cornwell, MD Previous PIs: Shaub Omer, MD Faisal Bakaeen, MD	Baylor College of Medicine Office of Research One Baylor Plaza, 600D, Houston, TX 77030	03-Jun-13	H-32818
699	Mount Sinai Beth Israel Beth Israel Medical Center 10 Nathan D. Perlman Pl, New York, NY 10003 Research office location: 350 E 17th St, 5th FL, New York, NY 10003	John Puskas**,** MD	Icahn School of Medicine at Mount Sinai One Gustave L. Levy Place, Box 1081, New York, NY 10029	28-Oct-14	HS #: 13-00479, GCO#1: 13-1233(0001)
573	Oklahoma Foundation for Cardiovascular Research Oklahoma Heart Hospital North 4050 W Memorial Rd, Oklahoma City, OK 73135 Research office location: 5200 e. I-240 Service Rd, Third Floor, Oklahoma City, OK 73135	Naeem Tahirkheli, MD Previous PI: Goya Raikar, MD	Western IRB 1019 39th Ave, SE Suite 120, Puyallup, WA 98374-2115	19-Mar-14 (MaGil IRB) 11-Sep-14 (WIRB)	WIRB protocol #: 20,130,138; Work Order #: 1-850949-1
674	Baylor College Medicine St Luke's Hospital One Baylor Plaza, MS-BCM390, Houston, TX 77030 Ben Taub General Hospital, 1504 Taub Loop, Houston, TX 77030	Todd Rosengart, MD	Baylor College of Medicine Office of Research One Baylor Plaza, 600D, Houston, TX 77030	04-Jun-13	H-32808
572	Instytutem Kardiologii im. Prymasa Tysiąclecia Stefana Kardynała Wyszyńskiego Alpejska 42 04-628 Warszawa, Poland	Prof Jacek Rozanski, MD	Bioethics Committee of Jagiellonian (Central Ethics Committee) University ul. Grzegorecka 20 PL 31-531 Krakow, Poland	29-Nov-12^1^ Ministry of Health Approval: 29-Aug-13	KBET/344/L/2012
636	The Heart Hospital Baylor Plano 4716 Alliance Blvd, Pavilion 2, Suite 310, Plano, TX 75093 Research office address: Baylor Research Institute 3310 Live Oak, Suite 501, Dallas, TX 75204	William Ryan, MD	Baylor Scott & White Research Institute 3310 Live Oak, Suite 501, Dallas, TX 75204	26-Jun-14	014-108, Reference #: 076,369
552	John Paul II Hospital Krakow Krakowski Szpital Specjalistyczny im. Jana Pawła II ul. Prądnicka 80, 31-202, Kraków, Poland	Prof Jerzy Sadowski, MD	Bioethics Committee of Jagiellonian (Central Ethics Committee) University ul. Grzegorecka 20 PL 31-531 Krakow, Poland	29-Nov-12 Ministry of Health Approval: 29-Aug-13	KBET/344/L/2012
532	University of Southern California Department of Cardiothoracic Surgery 1520 San Pablo St, Ste 4300, Los Angeles, CA 90033	Craig Baker, MD Previous PI: Vaughn Starnes, MD PhD	University of Southern California Health Sciences Campus IRB LAC + USC Medical Center General Hospital Suite 4700, 1200 N State St, Los Angeles, CA 90033	04-Mar-13	Project #: HS-13-00,059
183	New York Presbyterian Hospital – Columbia University Medical Center 177 Fort Washington Ave, Suite 7-435, New York, NY 10032	Hiroo Takayama, MD PhD Previous PI: Alan Stewart, MD	Columbia University IRB 154 Haven Ave, 1 Floor, New York, NY 10032	19-Nov-12	Protocol #: IRB-AAAK8159
715	North Mississippi Medical Center Inc 830 S Gloster St, Fourth Floor, East Tower, Tupelo, MS 38801 Research office address: Cardiology Associates Research LLC 830 S Gloster St, Third Floor, East Tower, Tupelo, MS 38801	David Talton, MD	North Mississippi Health Services IRB 830 S Gloster St, Tupelo, MS 38801	20-Jan-15	Not applicable
375	Spectrum Health Hospital 100 Michigan St, NE, Grand Rapids, MI 49503 Research office address: Spectrum Health System Cardiovascular Research, 330 Barclay Ave, NE, Suite GL1, Grand Rapids, MI 49503	Tomas Timek, MD Previous PI: Robert Hooker, MD	Spectrum Health Human Research Protection Program, Institutional Review Board 100 Michigan, NE, MC 038, Grand Rapids, MI 49503	09-Apr-13	IRB #: 2013-036
654	University of Kansas Hospital 3901 Rainbow Blvd, M/S 4035, Kansas City, KS 66160	George Trip Zorn, MD	University of Kansas Medical Center Human Research Protection Program 3901 Rainbow Blvd, M/S 1032, Kansas City, KS 66160	12-Jun-13	Project #: 13,709

*PI*, Principal investigator.
